# Aneurysmal Bone Cyst of the Sphenoid Body Mimicking Craniopharyngioma: A Case Report

**DOI:** 10.7759/cureus.23128

**Published:** 2022-03-13

**Authors:** Lauren J Pelkey, Bryan J Canty, Sean P Ferris, Devin T Mistry, Bryan E Figueroa

**Affiliations:** 1 Pathology, Ascension Health, Grand Township, USA; 2 Neuroradiology, University of Michigan Health-West, Wyoming, USA; 3 Department of Pathology, Division of Neuropathology, University of Michigan, Ann Arbor, USA; 4 Otolaryngology, University of Michigan, Metro Health Hospital, Grand Rapids, USA; 5 Neurosurgery, Great Lakes Neurosurgical Associates PC, Grand Rapids, USA

**Keywords:** craniopharyngioma, sphenoid sinus, sphenoid bone, sphenoid body, aneurysmal bone cyst

## Abstract

An aneurysmal bone cyst is a locally destructive benign lesion that predominately affects the long bones. Sphenoid body involvement is rare. To date, only 19 primary aneurysmal bone cysts of the sphenoid body have been reported. We describe the case of an 18-year-old male with a one-week history of severe right eye pain and lacrimation, right-sided diplopia, right-sided headache, photophobia, nausea, and vomiting. Magnetic resonance imaging (MRI) demonstrated a lobulated lesion centered in the sphenoid body with expansion into the cavernous sinus, sellar region, and clivus. The lesion had a homogenous hyperintense T2 signal with enhancing sidewalls and minimal septations. Computed tomography (CT) angiography revealed a hypoattenuating lesion containing a substance of nine Hounsfield units, compatible with water density. The clinicoradiologic findings were consistent with a craniopharyngioma. Intraoperatively, the lesion was confirmed to contain clear fluid and have prominent arterial feeding vessels. The extradural tumor was then excised with intralesional curettage. The histopathologic analysis resulted in a diagnosis of an aneurysmal bone cyst. This case highlights the potentially non-specific and variable appearance of aneurysmal bone cysts and the need to consider it in the differential diagnosis of sphenoid bone lesions.

## Introduction

An aneurysmal bone cyst is a benign osseous neoplasm characterized by rapid expansion and aggressive local growth. It accounts for 1-2% of all primary bone tumors [1]. The mean age of presentation is 18.4 years, with 80% of lesions occurring in individuals under 20 [2]. Lesions most frequently arise in the long bones, followed by the spine and flat bones of the pelvis [3]. Only 2-6% of aneurysmal bone cysts are found in the craniofacial bones, with the majority involving the calvarium [4]. Involvement of the sphenoid bone is rare.

Aneurysmal bone cysts are typically comprised of blood-filled spaces separated by connective tissue septa containing multinucleate osteoclast-type giant cells, reactive woven bone, and fibroblasts [5]. These lesions may be primary or secondary, with primary lesions accounting for 70% of cases [6]. Recent molecular data indicates primary aneurysmal bone cysts are of neoplastic origin, with approximately 75% of cases harboring ubiquitin carboxyl-terminal hydrolase 6 (USP6) gene translocations [7]. The neoplastic cells are bland stromal spindle cells, while the other cells present in these lesions are presumed to be reactive. Secondary aneurysmal bone cysts arise due to a primary lesion causing reactive aneurysmal bony expansion. These primary lesions include giant cell tumor of bone, fibrous dysplasia, chondroblastoma, osteoblastoma, and osteosarcoma [6]. 

Definitive diagnosis of an aneurysmal bone cyst requires exclusion of a primary lesion through histopathologic evaluation. However, radiologic studies may show highly suggestive findings. X-ray typically demonstrates a cystic and expansile osteolytic lesion with a soap-bubble appearance. CT often shows a radiolucent mass with localized bony erosion and thinned sidewalls. MRI classically reveals internal honeycomb septations with multiple fluid-fluid levels of different signal intensities. Fluid-fluid levels are a relatively specific finding for an aneurysmal bone cyst and are due to blood sedimentation of various ages [8]. When fluid-fluid levels occupy greater than two-thirds of the lesion, this finding is highly specific for an aneurysmal bone cyst [9]. Fluid-fluid levels may also occur in telangiectatic osteosarcomas, unicameral bone cysts, giant cell tumors, and chondroblastomas [9]. Additionally, the absence of fluid-fluid levels on MRI does not exclude a diagnosis of an aneurysmal bone cyst, as this finding may only be present in 30-44% of cases [10]. 

In this study, we review the literature on craniofacial aneurysmal bone cysts, discuss cases with atypical imaging features, and present an 18-year-old male with an aneurysmal bone cyst of the sphenoid body that mimicked a craniopharyngioma.

## Case presentation

An 18-year-old male presented with a one-week history of severe right eye pain and lacrimation, right-sided diplopia, right-sided headache, photophobia, nausea, and vomiting. The patient had a history of chronic sinusitis and denied recent head or facial trauma. The initial exam demonstrated right-sided diplopia but no cranial nerve palsies. Laboratory testing was normal.

**Figure 1:**
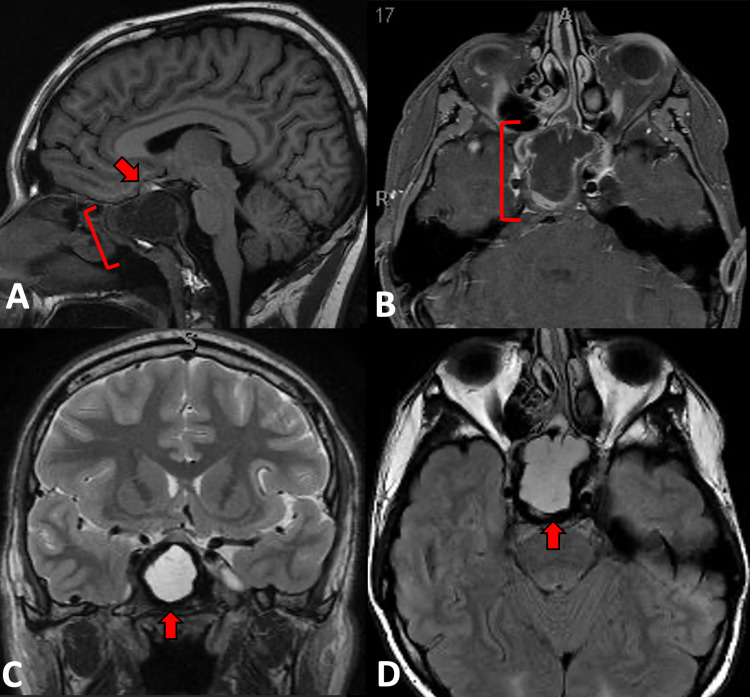
MRI Sagittal T1 (A) and axial (B) T1 post-contrast images reveal a hypointense partially septated expansile mass expanding the sphenoid sinus. Superior pituitary gland displacement (arrow on image A) can be appreciated. The mass has enhancing, nodular sidewalls (bracket on image B). Coronal T2 (arrow on image C) and FLAIR (arrow on image D) images show a hyperintense, multilobulated expansile lesion without fluid-fluid levels. FLAIR - fluid-attenuated inversion recovery

MRI revealed a cystic mass with superior displacement of the planum sphenoidale, pituitary gland, and infundibulum. There was expansion into the sella turcica and suprasellar cistern with enhancing sidewalls, lateral encroachment on the cavernous sinus, and destruction of the anterior clivus (Figure 1).

CT of the paranasal sinuses with contrast demonstrated a cystic, expansile lesion centered in the sphenoid body and sella turcica with peripheral enhancement. There was a breakdown of the posterior and right lateral walls of the sphenoid sinus and osseous remodeling of the clivus, dorsum sellae, and walls of the sphenoid sinus. There was leftward nasal septal deviation.

**Figure 2:**
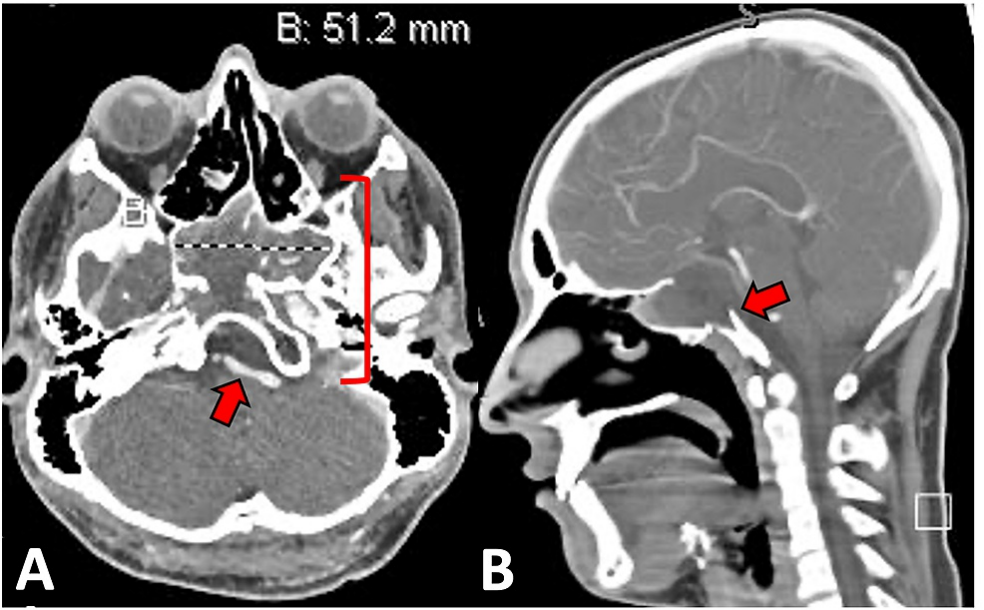
CT angiography Axial (A) and sagittal (B) CT angiogram demonstrate a large 5.1 x 4.7 x 3.0 cm (ML, AP, CC) hypodense lesion. The intratumoral contents contain a predominately homogenous substance of nine Hounsfield units. The lesion is eroding surrounding bone and expanding anteriorly near the sphenoethmoid junction, and inferiorly resulting in a bifid, eroded appearance of the clivus (arrow on image B). The cavernous (C4) and paraclinoid (C5) segments of the internal carotid artery appear as a curvilinear enhancement outlining the tumor margins (bracket on image A). The basilar artery appears as an "i" shaped enhancement anterior to the cerebellum (arrow on image A). ML - medial-lateral; AP - anterior-posterior; CC - cranial-caudal

CT angiogram revealed a large hypodense lesion filled with a predominately homogenous fluid of nine Hounsfield units, compatible with water density (Figure 2). Further, there was surrounding bony erosion and expansion anteriorly into the sphenoethmoid junction and inferiorly into the clivus. 

Nasal endoscopy demonstrated chronic pansinusitis, a deviated nasal septum with a hypertrophied nasal turbinate, and a sphenoid mass.

Based on the collective clinicoradiologic findings, the primary pre-operative diagnosis was a craniopharyngioma. Other considerations included a pituitary macroadenoma, primary sinus neoplasm, retention cyst, and mucocele.

Surgery was performed via an image-guided endoscopic endonasal transsphenoidal approach. Upon entering the sphenoid ostium, a fluctuant, cystic soft-tissue mass was encountered and emitted copious amounts of clear fluid and fresh blood. The tumor occupied the entire sphenoid sinus, obliterating the superior clivus and projecting into the posterior cranial fossa. A four-handed co-surgical approach was used to dissect the tumor in its capsule from the sphenoid sinus and from the anterior, middle, and posterior fossa dura. The mass was entirely extradural in nature. Complete enucleation of the cyst and all of its contents was achieved with curettage.

**Figure 3:**
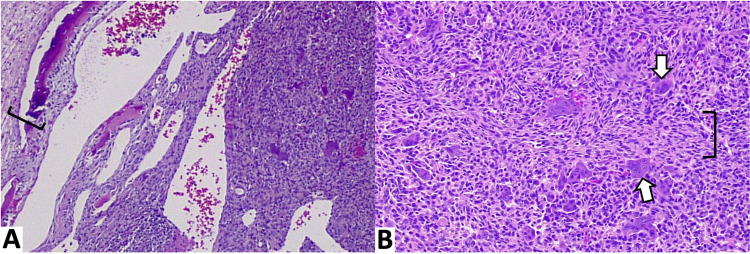
Histology of cystic and solid component Histologic sections demonstrate cystic and solid components with a peripheral osteoid rim (bracket on image A). A higher power view of the solid components of the tumor demonstrates frequent multinucleate, osteoclast-like giant cells (arrows on image B) with intervening spindled fibrous stroma (bracket on image B).

Histologic sections demonstrated a mixed cystic and solid tumor (Figure 3). Cystic blood-filled spaces were present with septa containing giant cells and spindled fibrous stroma without endothelial lining (Figure 3A). The central solid areas contained frequent osteoclast-like giant cells, which were diffusely spread throughout the tumor (Figure 3B). The giant cells uniformly contained less than 10 nuclei and were separated by mononuclear ovoid and spindled stromal cells along with numerous hemosiderin-laden macrophages and scattered lymphocytes (Figure 3B).

**Figure 4:**
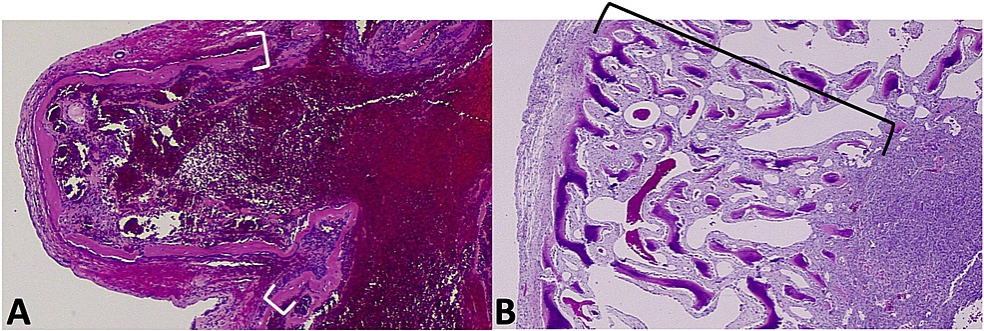
Histology of osteoid component The osteoid at the periphery of the tumor varied from areas with long linear deposits of immature osteoid with a buckled, crinkled appearance (brackets on image A) to areas with the rigid, trabecular arrangement, which was often calcified (bracket on image B).

Lace-like immature osteoid was located throughout the lesion with accentuation at the periphery of the tumor (Figure 4). The osteoid at the periphery of the tumor varied from areas with long linear deposits of immature osteoid with a buckled, crinkled appearance (Figure 4A) to areas with a rigid, trabecular arrangement, which was often calcified (Figure 4B). No malignant appearing osteoblasts, atypical stromal cells, or chondroid components were present. Additionally, there was no high mitotic activity nor atypical mitotic figures. A diagnosis of an aneurysmal bone cyst was made.

One month following surgery, the patient had complete resolution of chronic sinus issues, headaches, and diplopia. Due to the relatively high recurrence rate of aneurysmal bone cysts, follow-up will entail serial MRI every three months for one-year post-op, and then every six months for two to three years post-op [11].

## Discussion

Primary aneurysmal bone cysts involving the sphenoid body are rare, with only 19 cases reported in the literature [12-31]. Clinical presentation depends on tumor location and the degree of impingement of nearby skull base structures. Patients commonly present with pain and localized swelling secondary to rapid bony expansion. Clinical signs of a sphenoid bone aneurysmal bone cyst have included nasal septum deviation [13], facial asymmetry [32], hypernasal speech [33], hypertelorism [34], facial swelling [35], proptosis [21], ptosis [32], and strabismus [36]. Symptoms may include cranial nerve neuropathies [12], visual disturbances [16], sinus obstruction [18], purulent rhinitis [26], headache [15], anosmia [31], epistaxis [14], and nausea and vomiting [36].

Our patient presented with symptoms related to an expansile sphenoid mass. Though the pituitary gland was superiorly displaced by the tumor, there was no evidence of hypopituitarism. This lesion did not exhibit the classic radiologic findings of an aneurysmal bone cyst, as it lacked fluid-fluid levels. Internal septations were also minimal. A lesion with clear fluid and lack of septations is more consistent with a unicameral bone cyst. However, a unicameral bone cyst is non-expansile and would not be expected to grow outside the typical confines of the sphenoid bone [37].

Though aneurysmal bone cysts are typically filled with variably aged blood, they have also been found to contain xanthochromic serosanguineous fluid. Serous fluid with relevant impurities due to blood cells would be expected to be at least 40 Hounsfield units [38]. This is in contrast to our patient’s lesion, which contained a clear fluid density of nine Hounsfield units - a rarely reported phenomenon. 

However, unusual imaging findings may be expected in craniofacial aneurysmal bone cysts. Manoharan et al. described an aneurysmal bone cyst of the left zygoma in a 30-year-old female, which had evidence of a periosteal reaction and sunray pattern on cone-beam CT - a finding commonly described in malignant bone tumors [39, 40]. Kletke et al. discussed the case of a 23-year-old woman with an aneurysmal bone cyst of the middle cranial fossa with an enhancing “dural tail” on T1 post-contrast - a finding typically associated with meningiomas [41]. Shivakumar et al. reported an aneurysmal bone cyst of the sphenoethmoid sinus, appearing as a T1 isointense polypoid soft tissue mass that occluded the left nasal cavity and nasopharynx and was thought to be an angiofibroma [14]. Mihaylova et al. described an aneurysmal bone cyst in the root of a mandibular tooth in a 14-year-old male that appeared as an osteolytic unilocular cyst and mimicked an odontogenic cyst of the jaw [42].

Preoperatively, the clinicoradiologic features were most consistent with a craniopharyngioma, due to the cystic appearance, peripheral enhancement, and sellar location of the lesion [43]. Further, craniopharyngiomas have been found to be the most common tumor of the sellar region in childhood, accounting for 5.6-13% of all intracranial tumors within this population [44, 45]. Radiologically, they often follow the 90% rule, whereby 90% are predominantly cystic, 90% have enhancing cyst walls, and 90% have tumor calcifications [46]. However, any individual craniopharyngioma may exhibit all of these findings or none of them [47]. Classically, a craniopharyngioma has fluid-filled cysts containing blood products, cholesterol, and protein - a thick conglomerate resembling motor oil [46]. Histologic evaluation excluded the diagnosis of craniopharyngioma along with other lesions that can present similarly in this location, including pituitary adenoma, chondrosarcoma, chordoma, and mucocele. A secondary aneurysmal bone cyst was excluded by the lack of identification of another bone lesion.

Surgical intervention is the mainstay of treatment for an aneurysmal bone cyst. The two most common surgical techniques are wide en bloc excision and intralesional curettage with or without bone grafting. Wide en bloc excision requires removal of surrounding healthy tissue to ensure clear margins, while intralesional curettage involves entering the bony tumor and scooping out the contents with a curette. Intralesional curettage is preferred for lesions closely approximating neurovasculature, as in our patient’s case. For the majority of parasellar and clival tumors, the endonasal transsphenoidal approach is the standard procedure [48]. The overall prognosis is favorable. A 2021 review on the head and neck aneurysmal bone cysts reported a recurrence rate of 5.6%, most frequently arising within the year following surgery [49].

## Conclusions

Aneurysmal bone cysts can rarely occur in the sphenoid bone. These lesions typically appear as expansile radiolucent lesions with a surrounding thin shell of bone on CT. MRI may demonstrate internal honeycomb septations with fluid-fluid levels and heterogeneous signal intensity. However, classical imaging findings may be lacking when septations are minimal and variably aged blood is not present. This case highlights the potentially non-specific and variable appearance of an aneurysmal bone cyst occurring in this location and the need to consider it in the differential diagnosis of the sphenoid bone and parasellar lesions.
